# Association between Polymorphism of Interleukin-1beta and Interleukin-1 Receptor Antagonist Gene and Asthma Risk: A Meta-Analysis

**DOI:** 10.1155/2015/685684

**Published:** 2015-03-02

**Authors:** Yuanzhou He, Shuang Peng, Weining Xiong, Yongjian Xu, Jin Liu

**Affiliations:** Department of Respiratory Diseases, Tongji Hospital, Key Laboratory of Pulmonary Diseases of Health Ministry, Tongji Medical College, Huazhong University of Science and Technology, Wuhan 430000, China

## Abstract

*Background*. Asthma is a complex polygenic disease in which gene-environment interactions are important. A number of studies have investigated the polymorphism of IL-1*β* -511C/T and IL-1RA genes in relation to asthma susceptibility in different populations. However, the results of individual studies have been inconsistent. Accordingly, we conducted a comprehensive meta-analysis to investigate the association between the IL-1*β* -511C/T and IL-1RA polymorphism and asthma risk. *Methods*. Data were collected from the following electronic databases: Pub Med, China National Knowledge Infrastructure (CNKI), Chinese Biomedical Literature Database (CBM), ISI Web of Knowledge, and Google Scholar Search databases with the last report up to July 2013. Finally, 15 studies were included in our meta-analysis. We summarized the data on the association between IL-1*β* -511C/T and IL-1RA polymorphism and risk of asthma in the overall population and performed subgroup analyses by ethnicity, mean of age, and source of controls. Odds ratio (OR) and 95% confidence interval (CI) were used to evaluate the associations between IL-1*β* -511C/T and IL-1RA polymorphism and asthma risk. Statistical analysis was performed with Review Manager 5.1. *Results*. A total of 15 case-control studies were included in the meta-analysis of IL-1*β* -511C/T (1,385 cases and 1,964 controls) and IL-1RA (2,800 cases and 6,359 controls) genotypes. No association was found between IL-1*β* -511C/T polymorphism and asthma risk (dominant model: OR = 1.11, 95% CI: 0.99–1.25, *P* = 0.07, *P*_Heterogeneity_ = 0.06; recessive model: OR = 1.04, 95% CI: 0.91–1.20, *P* = 0.55, *P*_Heterogeneity_ = 0.11). Subgroup analysis based on ethnicity (Asian and Caucasian), source of controls (population-based controls and hospital-based controls), and mean of age (adulthood and childhood) did not present any significant association. The overall results showed that the IL-1RA polymorphism was related to an increased risk of asthma (homozygote model: OR = 1.32, 95% CI: 1.12–1.56, *P* = 0.0009, *P*_Heterogeneity_ = 0.87; recessive model: OR = 1.39, 95% CI: 1.18–1.63, *P* = 0.0001, *P*_Heterogeneity_ = 0.82). Similar results were found in the subgroup analyses by ethnicity, mean of age, and source of controls. Sensitivity analysis did not perturb the results. *Conclusions*. This meta-analysis provided strong evidence that the IL-1RA polymorphism was a risk factor of asthma, especially in Caucasian populations. However, no association was found for IL-1*β* -511C/T genotype carriers. Larger scale studies are needed for confirmation.

## 1. Introduction

Asthma is a common, chronic inflammatory disease of the airways that affects over 300 million individuals worldwide and is associated with 250,000 premature deaths each year [1–3]. In developed countries, the prevalence of asthma has increased considerably over the past three decades [4]. Currently, it is recognized that asthma is a multifactorial disease that results from complex interactions between environmental and genetic factors [5]. Factors that regulate the inflammation responses play an important role in the pathogenesis of asthma [6]. This inflammation is regulated by a number of various cytokines originating from inflammatory and structural tissue cells. Proinflammatory cytokines, a subgroup of the cytokines, could play a significant role in the pathomechanism of asthma [7–9]. In this regard, proinflammatory cytokines and their gene polymorphism seem to be important. Recent evidence indicates that gene variations of cytokines and their receptors are associated with the risk of asthma [10–12]. Two such candidate genes are interleukin-1beta (IL-1*β*) and interleukin-1 receptor antagonist (IL-1RA). The proinflammatory effect of IL-1*β* and the anti-inflammatory effect of IL-1RA in asthma have been documented in a number of human and animal studies [13–17].

Gene-by-environment factor seems to be a key process in the development and expression of asthma [18]. The airway inflammatory component of asthma is partly controlled by the genetic background of the patients [6]. Cytokine gene polymorphism could affect the serum levels of cytokine by influencing the transcriptional regulation. IL-1 is a major proinflammatory cytokine which could be seen in two forms of IL-1*α* and IL-1*β* [6, 7]. These molecules are structurally related and they share a similar profile of functions by binding to the same receptors with different affinity. The natural inhibitor of IL-1, IL-1 receptor antagonist, mediates its effect by binding IL-1 type I receptor and blocking IL-1 binding on target cells.

The IL-1 gene linkage to asthma has been reported in more than one study [19]. The human genes for IL-1*β*, IL-1*α*, their receptors, and the IL-1 receptor antagonist are clustered on chromosome 2 (q14–q21) [7, 20, 21]. The IL-1RA gene is part of the IL-1 gene and is structurally related to IL-1*β* with which it competes for occupancy of IL-1 cell surface receptors (IL-1RA) [22]. The IL-1RA polypeptide binds to the IL-1 cell receptors, in an attempt to reduce inflammatory responses [23]. The balance of cytokine production, receptor expression, and inhibitor levels seem to be a major factor in determining the outcome of the inflammatory response [24–26]. Constitutional polymorphisms in the IL-1 family of genes may lead to individual variations in cytokine secretion in qualitative terms [25–27].

From 1996 to 2013, merging studies have been done to evaluate the association between the IL-1*β* (-511C/T) and/or IL-1RA polymorphism and asthma risk in different populations [28–42]. Some of these studies have demonstrated a significant association of these two gene polymorphisms with asthma. However, the results were not consistent with other studies. Considering that a single study may lack the power of providing a reliable conclusion, we performed a meta-analysis to investigate the relationship between the IL-1*β* (-511C/T) and IL-1RA gene variants and asthma. To our knowledge, this is the first meta-analysis of the association between the IL-1*β* (-511C/T) and IL-1RA polymorphisms and asthma susceptibility.

## 2. Methods

### 2.1. Search Strategy

**Figure 1:**
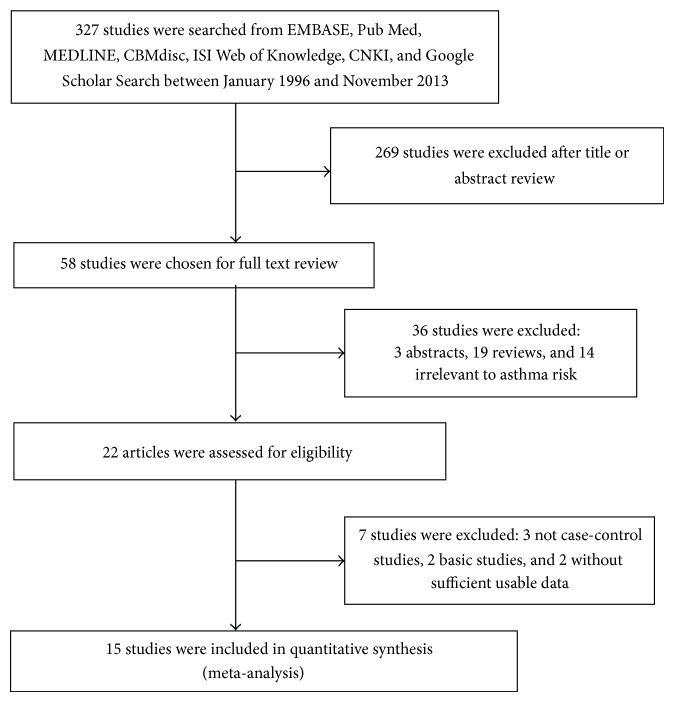
Flow of studies identification, inclusion, and exclusion.

All studies published between January 1996 and July 2013 that investigated the association between interleukin-1*β* (-511C/T) and interleukin-1 receptor antagonist polymorphisms with asthma risk were considered in the meta-analysis. We searched the Pub Med, CNKI (China National Knowledge Infrastructure), CBM (Chinese Bio-medicine Database), ISI web of knowledge, and Google scholar search by computer. The keywords used were as follows: asthma, asthma genetics, and interleukin-1*β* (-511C/T) and interleukin-1 receptor antagonist and polymorphism or variant or genotype without language restriction. Additional studies were identified by hands-on searches from references of original studies or review articles on this topic. For assessing association, human studies, regardless of sample size, were included if they met the following criteria: (1) interleukin-1*β* polymorphism at -511C/T and interleukin-1 receptor antagonist polymorphism at rs2234678 were determined. (2) Studies were case-control design (retrospective or nested case-control). (3) Each genotype frequency was reported, and there was enough information for extraction of data. (4) If studies had partly overlapped subjects, only the one with a larger and/or latest sample size was selected for the analysis. (5) Genotype distribution of control group must be consistent with Hardy–Weinberg equilibrium (HWE) by using genotype frequencies. Studies were excluded if one of the following existed: (1) not relevant to interleukin-1*β* (-511C/T) and interleukin-1 receptor antagonist or asthma, (2) the design based on family or sibling pairs, (3) genotype frequencies or number not reported, and (4) reviews and abstracts. For overlapping studies, only the one with the largest sample numbers was included (Figure 1).

### 2.2. Data Extraction

Information such as the first author, year of publication, country of origin, ethnicity, control source (population-based (PB), hospital-based (HB)), mean of age (adulthood, childhood), total number of cases and controls, and number of cases and controls with the wild-type, heterozygous and homozygous genotypes was collected from each study. Two investigators (Yuanzhou He and Shuang Peng) independently reviewed the papers to exclude irrelevant and overlapping studies. The results were compared, and disagreements were resolved by discussion and consensus. When overlapping papers were found, we only included the paper that reported the most extensive information.

### 2.3. Statistical Analysis

All statistical tests performed in this study were two-tailed and *P* values less than 0.05 were considered significant, unless otherwise stated. Statistical analyses were performed using Review Manager 5.1 Software (Nordic Cochrane Center, Copenhagen, Denmark). The strength of the association between the interleukin-1*β* (-511C/T) and interleukin-1 receptor antagonist gene polymorphism and asthma risk was measured by odds ratios (ORs) with their 95% confidence intervals (CIs). The statistical significance of the summary OR was determined with the *Z*-test (*P* < 0.05 was considered significant).

Heterogeneity between studies was assessed using *Q* test and *P* and *I* ^2^ value. *I* ^2^ was a value that could describe the percentage of variation across studies, where 0–25% indicated no observed heterogeneity and larger values showed increasing heterogeneity, with 25–50% regarded as low, 50–75% as moderate, and 75–100% as high. *P* > 0.05 for the *Q*-test indicated a lack of heterogeneity across studies which allowed the use of the fixed effects model; otherwise, the random effects model was used. The heterogeneity was adjusted by subgroup analysis. We investigated the association between the 2 genetic variants and asthma risk under homozygote and heterozygote comparisons and dominant and recessive genetic models. In addition, we conducted subgroup analyses by ethnicity, mean of age, and source of controls (population-based controls and hospital-based controls).

The publication bias of the selected studies was examined with funnel plots and further assessed using the tests of asymmetry of Begg and Mazumdar [43] and Egger et al. [44]. *P* values less than 0.05 were considered to be statistically significant in the meta-analysis.

## 3. Results

### 3.1. Study Inclusion

Main characteristics of the included publications investigating the association of interleukin-1*β* (-511C/T) and IL-1RA polymorphism with asthma are presented in Tables 1 and 2. There were 15 papers relevant to the searching words. The flow chart in Figure 1 summarizes this literature review process. In the current study, A total of 15 case-control studies were included in the meta-analysis of the -511C/T polymorphism in IL-1*β* (1,385 cases and 1,964 controls) and IL-1RA (2,800 cases and 6,359 controls) genotypes. Among the 15 studies, five studies contained two different variant gene data [28–31, 35]. All of the cases were confirmed as asthma. Controls were mainly healthy populations. Genotype distributions in the controls of all studies were in agreement with HWE.

### 3.2. Main Results

#### 3.2.1. Association between IL-1*β* -511C/T Polymorphism and Asthma Susceptibility

**Figure 2:**
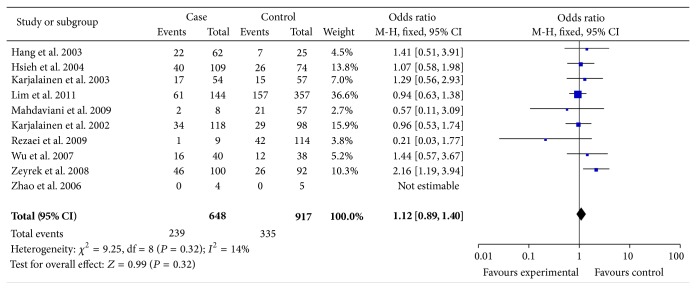
Forest plot the IL-1*β* -511C/T polymorphism and asthma risk (CC verses TT).

Totally, 10 studies met the inclusion criteria and were selected in the meta-analysis with 1,385 cases and 1,964 controls for analysis of the asthma risk and IL-1*β* -511C/T polymorphism.  Table 3 listed the main results of this meta-analysis. Overall, no significantly elevated asthma risk was associated with IL-1*β* -511C/T when all studies were pooled into the meta-analysis (show in the Table 3). In the subgroup analyses by mean of age and source of controls (population-based and hospital-based), no significant associations were found for all genetic models. The individual risk estimates were calculated and presented as forest plots (Figure 2).

#### 3.2.2. Association between IL-1RA Polymorphism and Asthma Susceptibility

**Figure 3:**
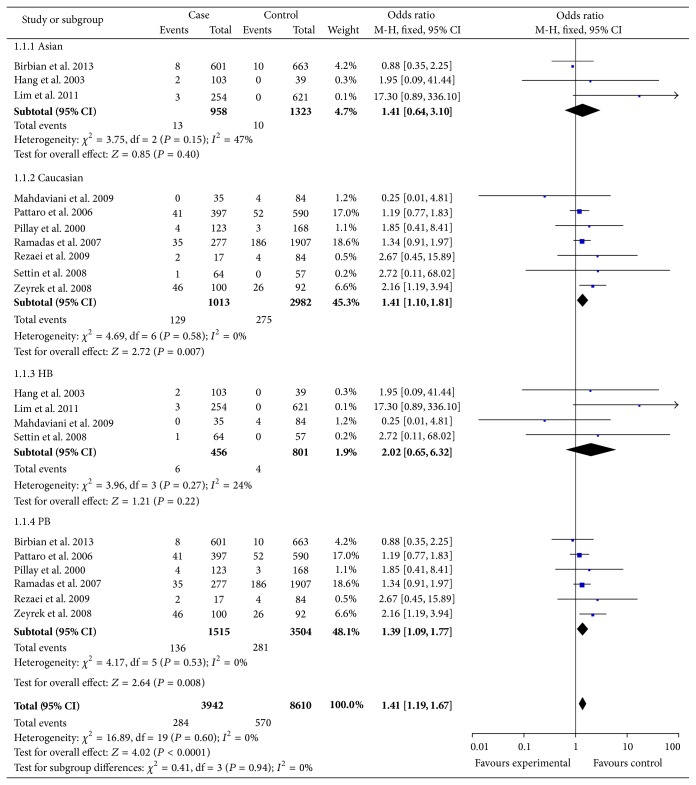
Forest plot the IL-1RA polymorphism and asthma risk (AA versus GG).

Data from 10 case-control studies comprising 2,800 cases and 6,359 controls were pooled together for analysis of the IL-1RA polymorphism. The overall data showed that the individuals who carried the IL-1RA genotype had a significantly increased asthma risk compared with those who carried the IL-1RA present genotype in all subjects (shown in the Table 4). When we considered the source of the control groups, high risks were found between asthma and IL-1RA polymorphism in population-based (AA versus GG: OR = 1.30, 95% CI: 1.12–1.56, *P* = 0.0009, *P*_Heterogeneity_ = 0.87; AA versus GA/GG: OR = 1.35, 95% CI: 1.07–1.70, *P* = 0.01, *P*_Heterogeneity_ = 0.75), but we did not find increased asthma risks with IL-1RA in hospital-based (AA versus GG: OR = 2.02, 95% CI: 0.65–6.32, *P* = 0.22, *P*_Heterogeneity_ = 0.27; AA versus GA/GG: OR = 2.71, 95% CI: 0.83–8.89, *P* = 0.10, *P*_Heterogeneity_ = 0.36). In the subgroup analyses by mean of age (adulthood and childhood), both of which increase asthma risk in any gene model (Figure 3).

### 3.3. Sensitivity Analysis and Publication Bias

The leave-one-out sensitivity analysis indicated that no single study changed the pooled ORs qualitatively (data not shown). The corresponding pooled ORs were not materially altered in all subjects and subgroups of IL-1*β* -511C/T and IL-1RA (data not shown). The results of sensitivity analyses indicated the stability of the results of this meta-analysis.

**Figure 4:**
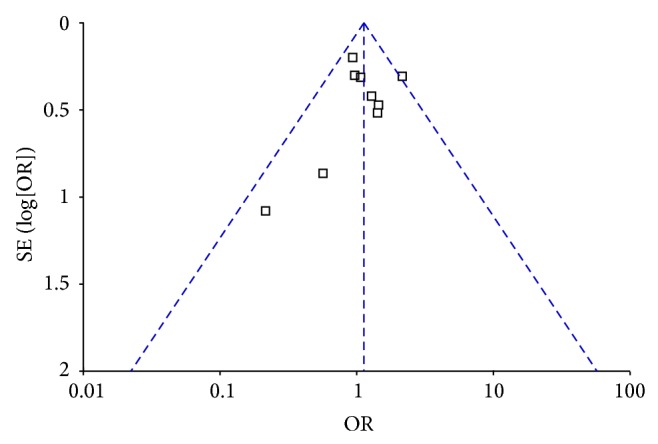
Funnel plots of IL-1*β* -511C/T polymorphism and asthma risk for publication bias. (CC versus TT).

Funnel plot and Egger's test were performed to assess the publication bias. The funnel plot shapes of IL-1*β* -511C/T and IL-1RA polymorphisms did not indicate any evidence of obvious asymmetry and the *P* values of Egger's test were 0.023 and 0.452, so the results showed no evidence of publication biases (Figure 4).

## 4. Discussion

The results from our meta-analysis indicate the lack of association between IL-1*β* -511C/T polymorphism and asthma susceptibility, whereas a significant increased risk existed between asthma and IL-1RA polymorphism.

For the IL-1*β* -511C/T polymorphism, we failed to find the association between asthma risk and the polymorphisms. It is known that the allele frequencies of cytokine genes are not equally distributed throughout the human population but follow diverse ethnic patterns; therefore, the subgroups according to ethnicity were performed. Our results indicated that no significant asthma risks of people with IL-1*β* -511C/T polymorphism are in all subjects. Furthermore, we also found that IL-1*β* -511C/T polymorphism has no risk of asthma susceptibility when stratified by control source. It is worth noting that, owing to limited number of studies, our results concerning subgroup analysis should be interpreted with caution.

Our result suggested that a significant increased risk existed between asthma and IL-1RA polymorphism. This association was very robust, which did not vary materially when the sensitivity analyses were performed. When analyzed based on ethnicity, Caucasian populations with all genotypes had a higher risk of asthma; in contrast, no association was found in Asian populations. This result demonstrates that Caucasian populations might be susceptible to asthma compared to Asians. However, the possible reason of the conflicting results could be the different genetic backgrounds and environment they exposed to. Additionally, as limited sample size may have not enough statistical power to detect a real effect or generate a fluctuated estimation, the small sample size of Asians in this meta-analysis should also be taken into consideration. Furthermore, we also showed that IL-1RA has strikingly increased the risk of asthma susceptibility when stratified by control source. However, we obtained the highest risk of asthma when we only considered the population-based controls. This may be because the hospital-based studies have inherent selection biases due to the fact that such controls may not be representative of the study population or the general population, particularly when the genotypes under investigation were associated with the disease-related conditions that the hospital-based controls may have. Thus, the using of proper and representative population-based control subjects is very important in reducing biases in such genotype association studies. For this result, the probable reason could be the selection bias. To be specific, the differences of selection criteria or selection chance between population-based and hospital-based controls may be the main reasons of the selection bias. However, the exact reason needs to be further confirmed. In the subgroup analyses by mean of age (adulthood and childhood), both of which increase asthma risk in any gene model.

Heterogeneity is a potential problem that might affect the interpretation of the results. Significance heterogeneity between the studies of this Meta analysis existed in all comparisons. Significant heterogeneity existed in overall comparisons in each genetic model. The observed heterogeneity could be attributed to differences in several factors such as ethnic variations, environmental factors, and methodological factors in design and conduct of the studies. After subgroup analysis by ethnic, the heterogeneity was effectively decreased in asthma patients. Therefore, it can be presumed that the relatively large heterogeneity mainly results from differences of atopic status. The leave-one-out sensitivity analysis would not have materially altered the results of this pooled analysis, indicating that our results were robust. The publication bias for the association between this polymorphism and cancer risk was not observed.

Several limitations of this meta-analysis should be summarized and addressed. Firstly, the sample size was still relatively small for some stratified analyses. Secondly, the numbers of child and adult populations are small in the subgroup analyses by mean of age. Thirdly, in the subgroup analysis by ethnicity, the number of studies and subjects analyzed for IL-1*β* -511C/T and IL-1RA were small, and the statistical power was so low that caution should be taken in interpreting these results. A further analysis would be detected if more precise studies were available, such as age, body mass index, and sex contained. In spite of these, our meta-analysis had some advantages. First, studies included in our present meta-analysis strictly met our selection criteria. Second, we did not detect any publication bias indicating that the whole pooled result may be unbiased.

In conclusion, this meta-analysis suggests that IL-1*β* -511C/T polymorphism may not contribute to asthma susceptibility. However, the IL-1RA polymorphism is significantly associated with higher asthma risk. These results could have been partly distorted by inadequate statistical power and small sample sizes. Therefore, further studies are required to clarify the potential gene-gene and/or gene-environment interactions between polymorphisms in the IL-1*β* -511C/T and IL-1RA gene and asthma.

## Figures and Tables

**Table 1:** Main data of all studies included in the meta-analysis for the -511C/T polymorphism in IL-1*β* gene.

Reference, year	Ethnicity	Source of control	Mean of age (years)	Cases/control	Cases			Control		
	(country)				TT	TC	CC	TT	TC	CC
Hang et al. 2003 [35]	Asian (Taiwan)	HB	27.4	116/47	40	54	22	18	22	7
Hsieh et al. 2004 [40]	Asian (Taiwan)	HB	10	202/144	69	93	40	48	70	26
Karjalainen et al. 2003 [38]	Caucasian (Finland)	PB	58	99/98	37	45	17	42	41	15
Lim et al. 2011 [28]	Asian (Singapore)	HB	63.5	299/716	83	155	61	200	359	157
Mahdaviani et al. 2009 [30]	Caucasian (Iran)	HB	17.9	40/140	6	52	2	36	82	21
Karjalainen et al. 2002 [39]	Caucasian (Finland)	PB	57.7	200/200	84	82	34	69	102	29
Rezaei et al. 2009 [29]	Caucasian (Iran)	PB	8.6	30/280	8	21	1	72	166	42
Wu et al. 2007 [41]	Asian (China)	HB	39.5	76/76	24	36	16	26	38	12
Zeyrek et al. 2008 [31]	Caucasian (Turkey)	PB	9.4	268/228	54	168	46	66	136	26
Zhao et al. 2006 [42]	Asian (China)	HB	5.9	55/35	4	51	0	5	30	0

HB: hospital-based case-control; PB: population-based case-control.

**Table 2:** Main data of all studies included in the meta-analysis for the polymorphism in IL-1RA gene.

Reference, year	Ethnicity	Source of control	Mean of age (years)	Cases/control	Cases			Control		
	(country)				GG	GA	AA	GG	GA	AA
Birbian et al. 2013 [37]	Asian (India)	PB	38.1	815/825	593	214	8	653	162	10
Hang et al. 2003 [35]	Asian (Taiwan)	HB	27.4	115/47	101	12	2	39	8	0
Lim et al. 2011 [28]	Asian (Singapore)	HB	63.5	294/710	251	40	3	621	89	0
Mahdaviani et al. 2009 [30]	Caucasian (Iran)	HB	17.9	37/140	35	2	0	80	56	4
Pattaro et al. 2006 [34]	Caucasian (Germany)	PB	23.6	604/905	356	207	41	538	315	52
Pillay et al. 2000 [36]	Caucasian (African)	PB	26	149/209	119	26	4	165	41	3
Ramadas et al. 2007 [33]	Caucasian (USA)	PB	10	419/3057	242	142	35	1721	1150	186
Rezaei et al. 2009 [29]	Caucasian (Iran)	PB	6.5	29/140	15	12	2	80	56	4
Settin et al. 2008 [32]	Caucasian (Egypt)	HB	7.5	70/98	63	6	1	57	41	0
Zeyrek et al. 2008 [31]	Caucasian (Turkey)	PB	9.4	268/228	54	168	46	66	136	26

HB: hospital-based case-control; PB: population-based case-control.

**Table 3:** Stratified analyses of the IL-1*β*-511C/T polymorphism on asthma risk in meta-analysis.

	*N* ^a^	Case/control	CC versus TT		TC versus TT		CC/TC versus TT		CC versus TC/CC	
			OR (95% CI)	*P* ^b^	OR (95% CI)	*P* ^b^	OR (95% CI)	*P* ^b^	OR (95% CI)	*P* ^b^
Total	**10**	**1385/1964**	**1.12 [0.96, 1.32]**	**0.15**	**1.10 [0.98, 1.21]**	**0.10**	**1.11 [0.91, 1.25]**	**0.06**	**1.10 [0.93, 1.31]**	**0.11**
Ethnicities										
Asian	5	748/1018	1.05 [0.78, 1.41]	0.77	1.02 [0.80, 1.29]	0.97	1.03 [0.82, 1.28]	0.96	1.03 [0.80, 1.33]	0.68
Caucasian	5	637/946	1.24 [0.88, 1.76]	0.11	1.19 [0.94, 1.52]	0.006^C^	1.20 [0.95, 1.52]	0.01^C^	1.06 [0.78, 1.44]	0.03^C^
Source of control										
PB	4	597/806	1.29 [0.90, 1.84]	0.81	1.14 [0.91, 1.43]	0.05	1.11 [0.86, 1.41]	0.05	1.04 [0.91, 1.20]	0.18
HB	6	788/1158	1.03 [0.77, 1.37]	0.81	1.07 [0.82, 1.38]	0.14	1.13 [0.91, 1.39]	0.26	0.95 [0.74, 1.22]	0.23
Mean of age										
Adult	6	830/1277	1.03 [0.78, 1.35]	0.86	1.04 [0.85, 1.28]	0.03^C^	1.05 [0.06, 1.27]	0.10	0.98 [0.77, 1.24]	0.24
Childhood	4	555/687	1.35 [0.90, 2.40]	0.05	1.24 [0.93, 1.66]	0.41	1.26 [0.95, 1.67]	0.30	1.24 [0.88, 1.75]	0.18

*N* ^a^: number of comparisons.

*P* ^b^: value of *Q*-test for heterogeneity test.

^C^Random effects model was used when *P* value for heterogeneity test <0.05; otherwise, fixed effects model was used.

**Table 4:** Stratified analyses of the IL-1RA polymorphism on asthma risk in meta-analysis.

	*N* ^a^	Case/control	AA versus GG		GA versus GG		AA/GA versus GG		AA versus GA/GG	
			OR (95% CI)	*P* ^b^	OR (95% CI)	*P* ^b^	OR (95% CI)	*P* ^b^	OR (95% CI)	*P* ^b^
Total	**10**	**2800/6359**	**1.32 [1.12, 1.56]**	**0.87**	**0.95 [0.88, 1.02]**	**0.001C**	**0.99 [0.92, 1.07]**	**0.01C**	**1.39 [1.18, 1.63]**	**0.82**
Ethnicities										
Asian	3	1224/1582	1.41 [0.64, 3.10]	0.15	1.32 [1.08, 1.60]	0.13	1.32 [1.09, 1.60]	0.27	1.33 [0.60, 2.91]	0.13
Caucasian	7	1576/4777	1.31 [1.03, 1.68]	0.87	0.81 [0.71, 0.93]	0.001^C^	0.88 [0.78, 1.00]	0.001^C^	1.39 [1.10, 1.77]	0.86
Source of control										
PB	6	2284/5364	1.30 [1.02, 1.65]	0.88	1.02 [0.91, 1.15]	0.01^C^	1.07 [0.95, 1.19]	0.11	1.35 [1.07, 1.70]	0.75
HB	4	516/995	2.02 [0.65, 6.32]	0.27	0.57 [0.41, 0.78]	0.0001^C^	0.64 [0.45, 0.83]	0.0001^C^	2.71 [0.83, 8.89]	0.36
Mean of age										
Adult	6	2014/2836	1.05 [0.81, 1.36]	0.83	1.17 [1.03, 1.34]	0.02^C^	1.16 [1.02, 1.32]	0.07	0.96 [0.76, 1.21]	0.22
Childhood	4	786/3523	1.45 [1.06, 1.97]	0.16	0.74 [0.37, 1.48]	0.0001^C^	0.76 [0.39, 1.49]	0.0001^C^	1.36 [1.01, 1.82]	0.75

*N* ^a^: number of comparisons.

*P* ^b^: value of *Q*-test for heterogeneity test.

^C^Random effects model was used when *P* value for heterogeneity test <0.05; otherwise, fixed effects model was used.

## Abbreviations

IL-1*β*: Interleukin-1beta
IL-1RA: Interleukin-1 receptor antagonist
SNPs: Single nucleotide polymorphisms
HWE: Hardy-Weinberg equilibrium
OR: Odds ratio
CI: Confidence interval
REM: Random effect model
FEM: Fixed effect model
HB: Hospital-based case-control
PB: Population-based case-control
*P*_Heterogeneity_: Value of *Q*-test for heterogeneity test.
